# An Updated Systematic Review and Meta-Analysis of Randomized Controlled Trials on Postoperative Antibiotic Administration After Cholecystectomy for Acute Mild to Moderate Cholecystitis

**DOI:** 10.7759/cureus.100903

**Published:** 2026-01-06

**Authors:** Danhui Heo, Prajna Wijaya, Nazlia H Latulumamina, Almunthir S Altobi, Abrar Hussein, Farkas Gyula

**Affiliations:** 1 Surgery, Albert Szent-Györgyi Medical School, University of Szeged, Szeged, HUN; 2 School of Medicine, University of Indonesia, Depok, IDN; 3 School of Medicine, University of Pattimura, Ambon, IDN; 4 Biotechnology, University of Nizwa, Nizwa, OMN; 5 Colorectal Surgery, Chelsea and Westminster Hospital NHS Foundation Trust, London, GBR

**Keywords:** antibiotics, cholecystectomy, cholecystitis, infection, post-operative

## Abstract

The role of extended postoperative antibiotic therapy after cholecystectomy for mild-to-moderate acute cholecystitis remains controversial. This systematic review and meta-analysis aimed to update the existing evidence by incorporating more recently published randomized controlled trials to evaluate whether postoperative antibiotics reduce infectious complications in this patient population.

A systematic search identified randomized controlled trials comparing postoperative antibiotics versus no postoperative antibiotics following cholecystectomy for mild-to-moderate acute cholecystitis. All included patients received standard pre- or perioperative antibiotic prophylaxis. Primary outcomes were overall surgical site infection, superficial infection, deep wound infection, and organ space infection. Secondary outcomes included postoperative hospital stay, fever lasting more than two days, morbidity, and urinary tract infection. Five randomized controlled trials involving 931 patients were included, of whom 449 (48.2%) received postoperative antibiotics. Postoperative antibiotics did not significantly reduce overall surgical site infection rates or superficial and deep wound infections. A significant reduction in organ space infection was observed. No significant differences were found in postoperative hospital stay, postoperative fever, morbidity, or urinary tract infection.

Routine postoperative antibiotic administration after cholecystectomy for mild-to-moderate acute cholecystitis does not provide a significant benefit in reducing overall surgical site infections or other postoperative outcomes, apart from a limited reduction in organ space infections. When adequate source control is achieved with appropriate pre- or perioperative prophylaxis, a selective and patient-specific approach to postoperative antibiotic use is supported to minimize unnecessary antibiotic exposure and promote antimicrobial stewardship.

## Introduction and background

Acute cholecystitis, often caused by gallstones, is a significant health concern. Gallstones affect approximately 6.1% of the global population, with their incidence strongly linked to metabolic dysfunction and aging, both of which are on the rise [1-3]. The Tokyo Guidelines 2018 (TG18) recommend early laparoscopic cholecystectomy (LC) as the standard treatment for mild-to-moderate acute cholecystitis (Grades I and II) [4]. However, acute cholecystitis or biliary obstruction significantly increases the risk of surgical site infection (SSI), with a 58% higher risk in laparoscopic procedures compared to cases without these conditions, and a more than fourfold increased risk in open surgeries [5,6]. Postoperative infection rates for mild-to-moderate acute cholecystitis range from 7.6% to 12%, depending on patient factors, and SSIs not only prolong hospital stays but also impose substantial economic costs [7-9]. TG18 recommends tailoring antimicrobial therapy to the severity of acute cholecystitis, suggesting discontinuation within 24 hours postoperatively for Grades I and II cases if no evidence of infection persists [10]. Similarly, the 2020 World Society of Emergency Surgery guidelines advise against routine postoperative antibiotics when the infection focus is controlled, though this recommendation was based on limited evidence from a single randomized controlled trial (RCT), highlighting the need for further validation [11].

The efficacy of postoperative antibiotics in preventing infections remains debated. A 2019 meta-analysis by La Regina *et al. *found no significant reduction in SSIs with extended antibiotic use, though it was limited by the inclusion of only three studies [12]. Recent randomized controlled trials, such as the CHART trial (2018) and Zabihi-Mahmoudabadi et al. (2022), offer new insights [13,14]. This updated meta-analysis should incorporate recent randomized controlled trials and examine a broad range of both infectious and non-infectious outcomes, offering a more detailed perspective on the risks and benefits of postoperative antibiotics for these patients. While current guidelines and studies provide some insight, the use of antibiotics after cholecystectomy in mild-to-moderate acute cholecystitis remains a debated topic. Therefore, integrating the latest research to assess the full spectrum of outcomes is crucial to resolve ongoing controversies and support evidence-based clinical decisions.

This article was previously presented as a meeting abstract at the ASiT 49th Annual Surgical Conference, held from March 7th to 9th, 2025, organized by the Association of Surgeons in Training.

## Review

Methods

This systematic review and meta-analysis followed the guidelines outlined in the Cochrane Handbook for Systematic Reviews of Interventions (version 6.4) and adhered to the PRISMA (Preferred Reporting Items for Systematic Reviews and Meta-Analyses) Statement [15,16]. The meta-analysis was also prospectively registered in PROSPERO with the unique identifier CRD42024566682.

Eligibility Criteria

Studies were included in this meta-analysis if they met all of the following criteria: patients underwent cholecystectomy for mild to moderate acute cholecystitis; all patients received standard preoperative or perioperative antibiotic prophylaxis; the intervention group received extended postoperative antibiotic therapy, defined as continuation of antibiotics beyond the immediate perioperative period (≥24 hours after surgery); the comparator group received no postoperative antibiotics, including placebo or discontinuation of antibiotics after perioperative prophylaxis; and the study was a randomized controlled trial. Studies not published in English were excluded.

Definitions

In this systematic review, the severity of acute cholecystitis was classified using the Tokyo Guidelines 2018, which categorize the disease into three levels of severity based on clinical findings, laboratory results, and imaging characteristics.

Grade I (mild) acute cholecystitis describes cases without organ dysfunction and without marked local inflammatory changes. Grade II (moderate) acute cholecystitis is characterized by marked local inflammation, including elevated white blood cell count (>18,000/µL), a palpable tender mass in the right upper quadrant, symptom duration >72 hours, or imaging findings indicating severe local inflammation. Patients with Grade III (severe) acute cholecystitis, characterized by the presence of organ dysfunction, were excluded from this analysis. From this review, only studies that included patients with Tokyo Guidelines Grade I or II acute cholecystitis were considered eligible.

Perioperative antibiotic prophylaxis refers to antibiotic administration given before skin incision and/or within 24 hours after surgery, in accordance with standard surgical prophylaxis practices. Extended postoperative antibiotic therapy refers to continued antibiotic administration beyond 24 hours after cholecystectomy, typically for 3-7 days, as defined by the individual trial protocols.

Search Strategy

We conducted a systematic search of PubMed, EMBASE, ClinicalTrials.gov, and the Cochrane Library up to 15 July 2024. After duplicate removal, two reviewers (DH and PW) screened titles and abstracts and independently evaluated full-text articles for eligibility based on predetermined criteria. We applied backward snowballing by examining references in systematic reviews identified through our search terms. Disagreements were resolved by consensus. The search strategy included keywords such as “antibiotic,” “postoperative,” and “cholecystitis”.

Endpoints

Primary endpoints were SSI, superficial infection, deep wound infection, and organ space infection. Secondary endpoints were postoperative hospital stay, fever (>2 days), morbidity, and urinary tract infection.

Statistical Analysis

Risk ratios with 95% confidence intervals (CIs) were calculated to compare binary outcomes between the intervention and control groups. For continuous outcomes measured on the same scale across studies, mean differences were calculated using the inverse-variance method under a random-effects model. Statistical heterogeneity was assessed using the Cochran Q test and the I² statistic, with low heterogeneity defined as p> 0.10 and I²< 25%. A Mantel-Haenszel random-effects model was applied for dichotomous outcomes, and p-values ≤ 0.05 were considered statistically significant. All analyses were performed using Review Manager version 5.4 (The Cochrane Collaboration, Copenhagen, Denmark). When continuous outcomes were reported as medians rather than means, appropriate statistical methods were used to estimate means and standard deviations prior to pooling.

Sensitivity Analysis

Due to the limited number of studies, conducting a funnel plot analysis for publication bias was not possible. Instead, a sensitivity analysis using the “leave-one-out” method was conducted. In this approach, each study was sequentially excluded, and the pooled effect sizes were recalculated based on the remaining studies in the analysis.

Quality Assessment and Publication Bias

The included studies were evaluated for methodological quality using the Cochrane risk-of-bias tool for randomized trials (ROB-2), which assesses quality based on responses to signaling questions, judgments within each bias domain, and an overall risk-of-bias rating [17]. Two independent reviewers (PW and NL) conducted this assessment utilizing Cochrane’s macro-enabled Excel tool, resolving any disagreements through consensus. To reduce publication bias, we also searched for unpublished studies in ClinicalTrials.gov and the Cochrane database.

Results

Study Selection and Characteristics

**Figure 1:**
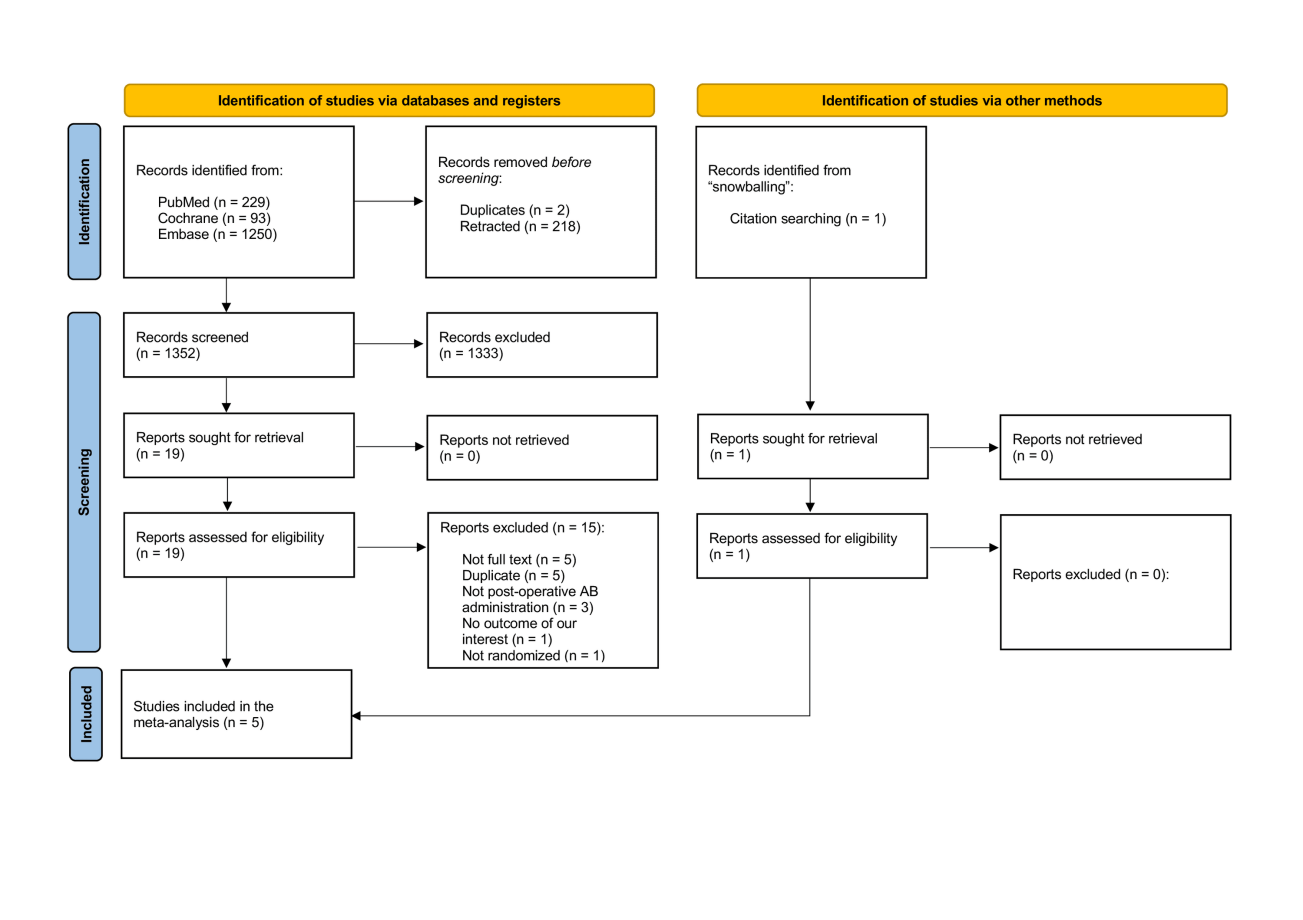
Flow Diagram for Selection of Relevant Clinical Studies.

As detailed in Figure 1, 1572 studies were identified using the search strategy in the databases and registry. After removing duplicate articles and unrelated studies as well as retrieving full paper, 19 were thoroughly reviewed for the inclusion and exclusion criteria. 5 studies [13,14,18-20] and 931 patients were included, of whom 449 (48.2%) received postoperative antibiotics. Population characteristics are presented in Table 1.

**Table 1:** Baseline Characteristics of the Included Studies.

Study	Cholecystitis grade (Tokyo guideline)	Preoperative or perioperative antibiotic	Postoperative antibiotic	Postoperative antibiotic group/cohort size	Type of cholecystectomy (n)
Zabihi-Mahmoudabadi et al., (2022) [14]	1-2	Ceftriaxone 1 g IV and metronidazole 500 mg IV	Ciprofloxacin and metronidazole, administered orally for 5 days	30/60	Laparoscopic (37), Open (23)
de Santibañes et al., (2018) [13]	1-2	Ampicillin/sulbactam IV every 6 hours until surgery	Amoxicillin/clavulanic acid (AMC) 1000 mg orally every 8 hours for 5 days	91/195	Laparoscopic (195)
Kim et al., (2017) [18]	1-2	1.0 g of 2nd-generation cephalosporin (cefoxitin sodium) IV, 3 times daily from diagnosis + single dose of the same antibiotics 30 min before surgery	Cefotixin 1 g IV, 3 times daily, then switch to cefaclor 250 mg orally, twice daily (when the patient began to eat) throughout hospitalization until discharge	93/188	Laparoscopic (188)
Loozen et al., (2017) [19]	1-2	Cefazolin 2g IV single dose, 15-30 min before surgery	Cefuroxime 750 mg IV + Metronidazole 500 mg IV, 3 times daily for 3 days	77/150	Laparoscopic (150)
Regimbeau et al., (2014) [20]	1-2	Amoxicillin/clavulanic acid 2 g IV, 3 times daily before surgery + once at the time of surgery	Amoxicillin/clavulanic acid 2 g orally, 3 times daily for 5 days	158/338	Laparoscopic (293), Conversion (27), laparotomy (18)

Quality Assessment

**Figure 2:**
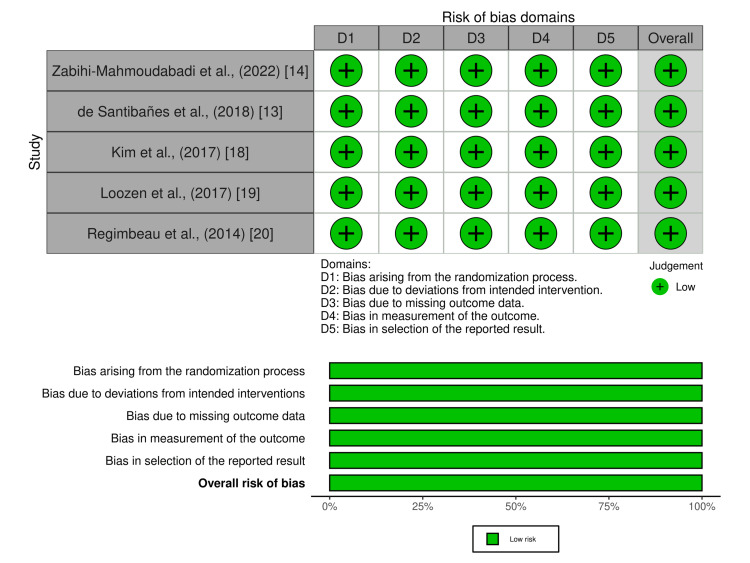
Risk of Bias Analysis.

Overall, all of the included studies demonstrated a low risk of bias across all domains. The assessment of the individual study is included in Figure 2.

Primary Outcomes

**Figure 3:**
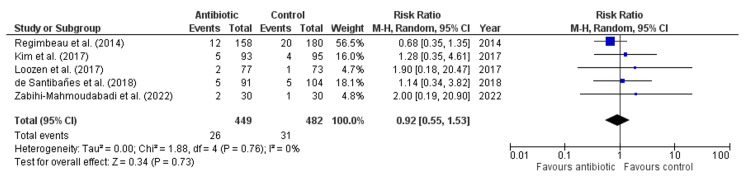
Forest Plot for Surgical Site Infection. Regimbeau et al., (2014) [20], Kim et al., (2017) [18], Loozen et al., (2017) [19], de Santibañes et al., (2018) [13], Zabihi-Mahmoudabadi et al., (2022) [14].

**Figure 4:**
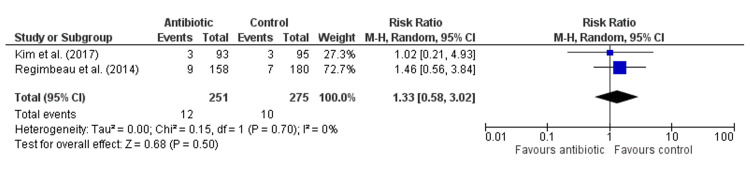
Forest Plot for Superficial Infection. Kim et al., (2017) [18], Regimbeau et al., (2014) [20].

**Figure 5:**
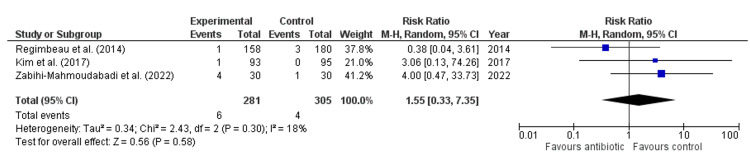
Forest Plot for Deep Wound Infection. Regimbeau et al., (2014) [20], Kim et al., (2017) [18], Zabihi-Mahmoudabadi et al., (2022) [14].

**Figure 6:**
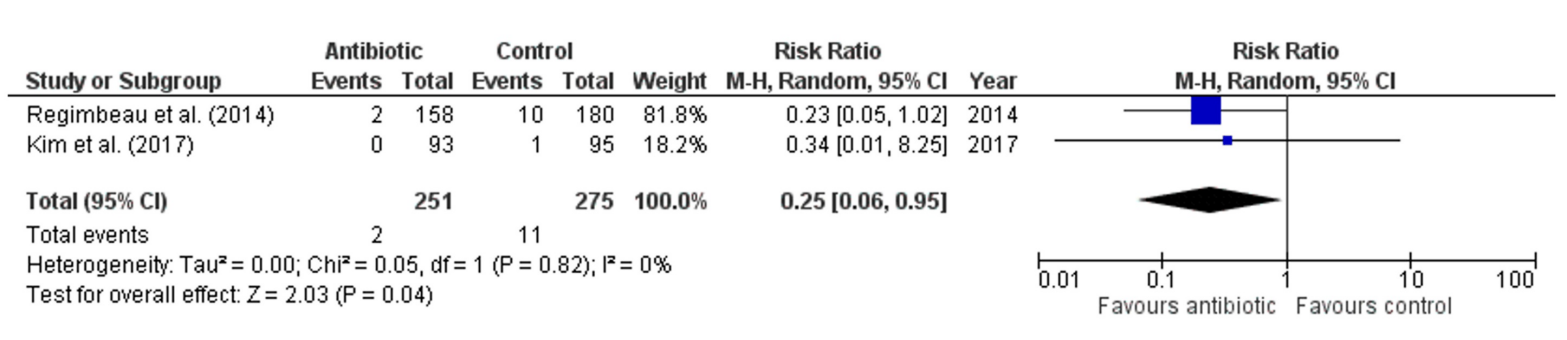
Forest Plot for Organ Space Infection Regimbeau et al., (2014) [20], Kim et al., (2017) [18]

Antibiotics did not significantly reduce the risk of SSI compared to control (Risk Ratio: 0.92, 95% CI: 0.55-1.53, p = 0.73, Figure 3), with no heterogeneity among studies (I² = 0%), indicating consistent findings. Similarly, antibiotics had no significant effect on the risk of superficial infection (Risk Ratio: 1.33, 95% CI: 0.58-3.02, p = 0.50, Figure 4), with an absence of heterogeneity (I² = 0%). For deep wound infection, antibiotics also showed no significant reduction in risk compared to control (Risk Ratio: 1.55, 95% CI: 0.33-7.35, p = 0.58, Figure 5), with low heterogeneity (I² = 18%). However, antibiotics did significantly reduce the risk of organ space infection compared to control (Risk Ratio: 0.25, 95% CI: 0.06-0.95, p = 0.04, Figure 6), with no heterogeneity observed (I² = 0%), indicating consistency across studies.

Secondary Outcomes

**Figure 7:**
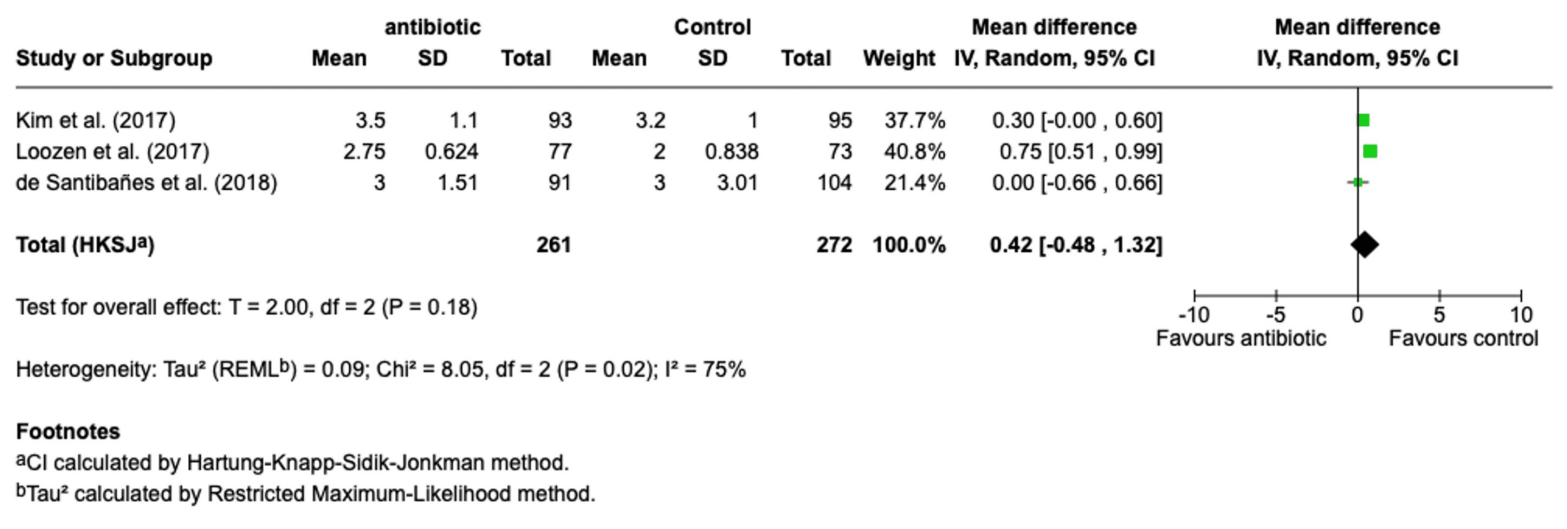
Forest Plot for Postoperative Hospital Stay. Kim et al., (2017) [18], Loozen et al., (2017) [19], de Santibañes et al., (2018) [13].

**Figure 8:**
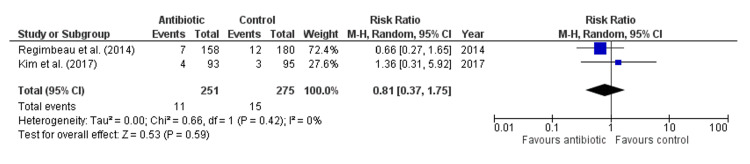
Forest Plot for Fever (>2 days). Regimbeau et al., (2014) [20], Kim et al., (2017) [18].

**Figure 9:**
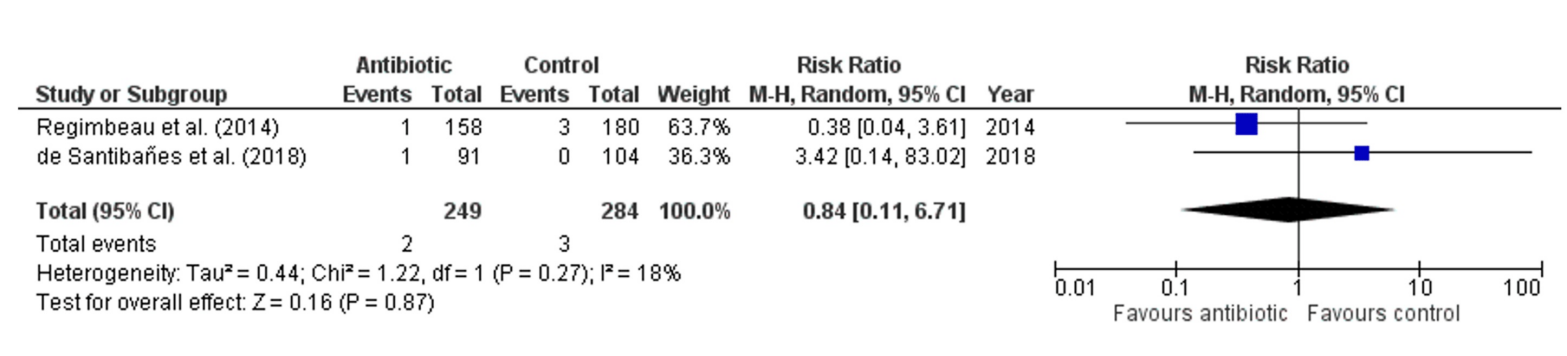
Forest Plot for Morbidity. Regimbeau et al., (2014) [20], de Santibañes et al., (2018) [13].

**Figure 10:**
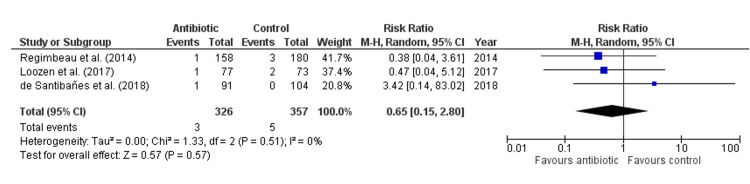
Forest Plot for Urinary Tract Infection. Regimbeau et al., (2014) [20], Loozen et al., (2017) [19], de Santibañes et al., (2018) [13].

Antibiotic treatment did not significantly reduce the length of postoperative hospital stay compared to control (Mean Difference: 0.42 days, 95% CI: -0.48 to 1.32, p = 0.18, Figure 7), with high heterogeneity observed across studies (I² = 75%), indicating substantial variability. Similarly, antibiotics did not significantly lower the risk of fever lasting more than two days (Risk Ratio: 0.81, 95% CI: 0.37-1.75, p = 0.59, Figure 8), with no heterogeneity detected (I² = 0%). There was also no significant effect of antibiotics on morbidity (Risk Ratio: 0.84, 95% CI: 0.11-6.71, p = 0.87, Figure 9), with low heterogeneity observed (I² = 18%). Lastly, antibiotics did not significantly reduce the risk of urinary tract infection (Risk Ratio: 0.65, 95% CI: 0.15-2.80, p = 0.57, Figure 10), with no heterogeneity among studies (I² = 0%), suggesting consistent findings across studies.

Sensitivity Analysis

A leave-one-out sensitivity analysis was performed for the primary outcome (surgical site infection). The overall conclusion remained unchanged, with p-values ranging from 0.47 to 0.73 and heterogeneity remaining at 0% across all cases, indicating that no single study disproportionately influenced the pooled result (Appendix Figure 11-15). 

For the secondary outcome of postoperative hospital stay, substantial heterogeneity was observed; therefore, a sensitivity analysis was performed. Across the leave-one-out analyses, heterogeneity ranged from 0% to 81%, and the pooled effect remained statistically non-significant in all cases. Notably, exclusion of the study by Loozen et al. [19], resulted in a marked reduction in heterogeneity to 0%, with no significant change in the overall effect estimate (p = 0.27) (Appendix Figures 16-18).

Discussion

In this systematic review and meta-analysis of five randomized controlled trials including 931 patients, we compared outcomes between patients undergoing cholecystectomy for mild to moderate acute cholecystitis who received extended postoperative antibiotic therapy and those who did not. The principal findings indicate that extended postoperative antibiotic use does not confer a meaningful reduction in overall surgical site infection, postoperative hospital stay, or overall morbidity when appropriate perioperative prophylaxis and effective surgical source control are achieved.

Specifically, extended postoperative antibiotic therapy was not associated with a reduction in overall surgical site infection, superficial infection, or deep wound infection. Although a statistically significant reduction in organ space infection was observed, this finding should be interpreted cautiously. Organ space infections were infrequent across the included trials, and the absolute number of events was low, limiting the robustness and clinical generalizability of this result. Consistent with this interpretation, Loozen et al. reported an infectious complication rate of only 4% in both the extended and single-dose prophylaxis groups, suggesting limited clinical relevance of prolonged antibiotic administration in the setting of low baseline infection risk. Although the non-inferiority of single-dose prophylaxis compared with extended regimens cannot be definitively established, the available evidence suggests that extended antibiotic prophylaxis offers minimal additional benefit in uncomplicated cases [19].

Laparoscopic cholecystectomy remains the gold standard treatment for mild to moderate acute cholecystitis [21,22]. While there is broad consensus regarding the initiation of preoperative or perioperative antibiotic therapy when infection is suspected, the role of extending antibiotics postoperatively has been less clearly defined. The findings of the present study align with previous randomized trials and earlier meta-analyses demonstrating no significant reduction in surgical site infection with postoperative antibiotic continuation [12]. By incorporating additional randomized controlled trials beyond those included in earlier analyses, this updated meta-analysis strengthens the evidence base supporting a restrictive approach to postoperative antibiotic use in this population.

Variability in antibiotic regimens across studies reflected differences in local institutional guidelines rather than systematic differences in patient risk. For example, de Santibañes et al. employed a double-blind design comparing a β-lactam/β-lactamase inhibitor regimen with placebo, with high protocol adherence and complete follow-up at 7 and 30 days [13]. Despite methodological rigor and prolonged antibiotic exposure, no reduction in postoperative surgical site infection was observed. Similarly, although Zabihi-Mahmoudabadi et al. applied a different postoperative antibiotic strategy, outcomes were comparable, further suggesting that extended postoperative antibiotic therapy does not meaningfully influence infection rates in uncomplicated mild to moderate disease [14].

Current international guidelines, including the Tokyo Guidelines and recommendations from the Surgical Infection Society and the Infectious Diseases Society of America, emphasize that antibiotic selection and duration should be guided by disease severity and adequacy of source control [23,24]. In uncomplicated mild or moderate acute cholecystitis, postoperative antibiotics are generally not recommended once effective source control has been achieved. In contrast, extended postoperative antibiotic therapy is reserved for patients with moderate acute cholecystitis complicated by severe local inflammatory findings, such as gallbladder perforation, emphysematous changes, or necrosis, or for those with severe acute cholecystitis. The results of the present analysis are consistent with these recommendations, supporting selective rather than routine postoperative antibiotic use.

Consistent with guideline-based management, extended postoperative antibiotics in this analysis did not reduce the risk of deep infection, urinary tract infection, postoperative fever, or overall morbidity. These findings reinforce the concept that preoperative prophylaxis followed by timely surgical removal of the infectious focus is sufficient in uncomplicated Grade I-II acute cholecystitis. Avoiding unnecessary antibiotic exposure may reduce adverse drug events, limit the development of antimicrobial resistance, and decrease the incidence of *Clostridioides difficile* infection [25].

Length of hospital stay represents an important outcome with implications for healthcare utilization and cost. Although postoperative antibiotics did not significantly affect hospital stay in this analysis, substantial heterogeneity was observed. Sensitivity analyses suggested that this variability was likely driven by differences in surgical timing, perioperative optimization, institutional logistics, and antibiotic administration routes rather than by antibiotic use itself. Early laparoscopic cholecystectomy has consistently been shown to reduce morbidity, hospital stay, and healthcare costs compared with delayed surgery [26,27], and prolonged postoperative antibiotic administration appears unlikely to meaningfully influence these outcomes.

This meta-analysis has several strengths, including exclusive inclusion of randomized controlled trials, adherence to PRISMA methodology, and incorporation of more recent trials compared with earlier reviews. Nevertheless, limitations should be acknowledged. The number of included studies remains modest, antibiotic regimens and surgical techniques varied across trials, and some outcomes were limited by low event rates. Additionally, exclusion of non-English studies may introduce language bias and limit generalizability.

Future research should prioritize large, multicenter randomized controlled trials focusing on patients undergoing laparoscopic cholecystectomy for uncomplicated Grade I-II acute cholecystitis, which comprises the majority of cases in this analysis. Further investigation is warranted to clarify whether specific subgroups, such as patients with significant comorbidities or those undergoing primary open cholecystectomy, may benefit from extended postoperative antibiotic therapy. Studies evaluating optimal antibiotic duration, selection, antimicrobial resistance patterns, and cost-effectiveness will be essential to inform future clinical guidelines and healthcare policy.

## Conclusions

This systematic review and meta-analysis demonstrate that routine administration of postoperative antibiotics after cholecystectomy for mild-to-moderate acute cholecystitis does not significantly reduce surgical site infections or improve other postoperative outcomes. A limited benefit was observed in reducing organ space infections; however, the overall evidence does not support the routine use of postoperative antibiotics when appropriate pre or perioperative prophylaxis has been administered. These findings endorse a selective, patient-tailored approach to antibiotic stewardship, which may reduce unnecessary antibiotic exposure and associated risks. Future multicentre trials are needed to identify subgroups that may benefit from extended antibiotic therapy and to assess cost-effectiveness and resistance outcomes.

## Figures and Tables

**Figure 11:**
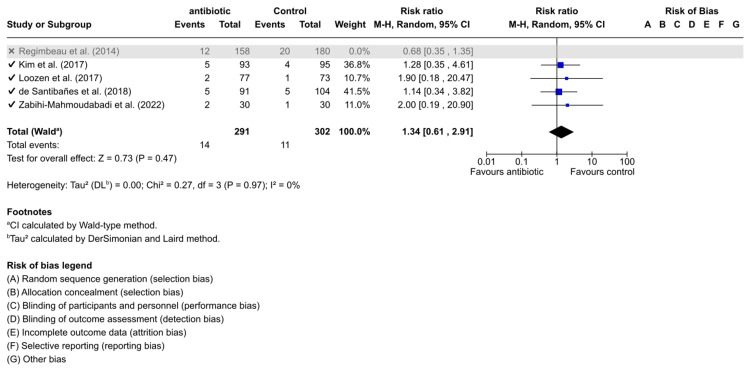
Sensitivity Analysis Results 1 (1). Regimbeau et al., (2014) [20], Kim et al., (2017) [18], Loozen et al., (2017) [19], de Santibañes et al., (2018) [13], Zabihi-Mahmoudabadi et al., (2022) [14].

**Figure 12:**
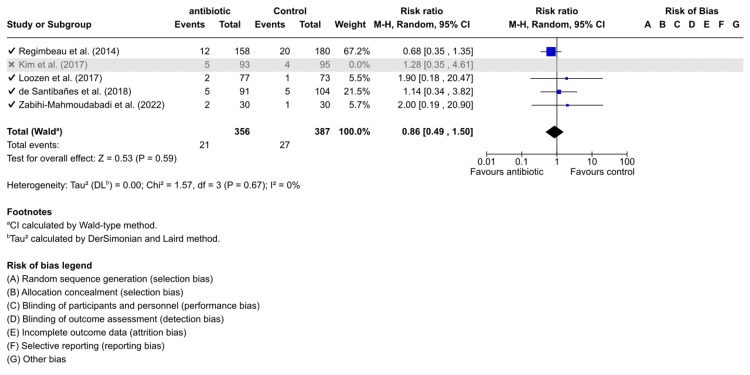
Sensitivity Analysis Results 1 (2). Regimbeau et al., (2014) [20], Kim et al., (2017) [18], Loozen et al., (2017) [19], de Santibañes et al., (2018) [13], Zabihi-Mahmoudabadi et al., (2022) [14].

**Figure 13:**
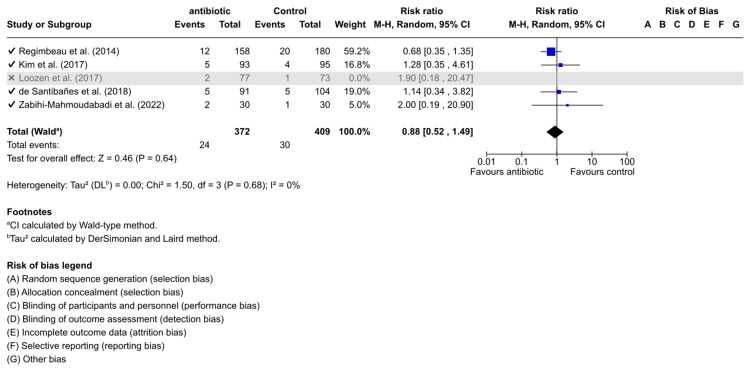
Sensitivity Analysis Results 1 (3). Regimbeau et al., (2014) [20], Kim et al., (2017) [18], Loozen et al., (2017) [19], de Santibañes et al., (2018) [13], Zabihi-Mahmoudabadi et al., (2022) [14].

**Figure 14:**
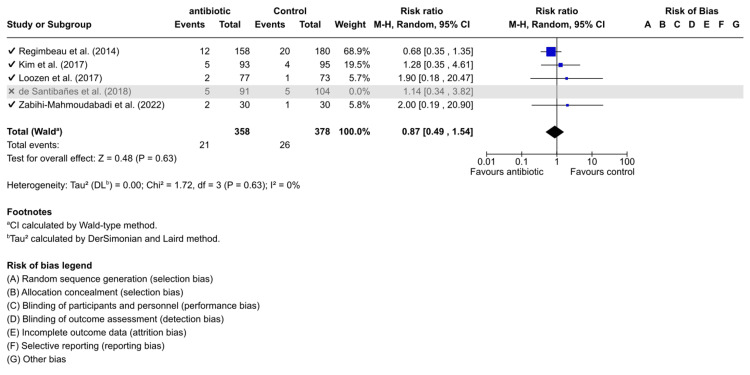
Sensitivity Analysis Results 1 (4). Regimbeau et al., (2014) [20], Kim et al., (2017) [18], Loozen et al., (2017) [19], de Santibañes et al., (2018) [13], Zabihi-Mahmoudabadi et al., (2022) [14].

**Figure 15:**
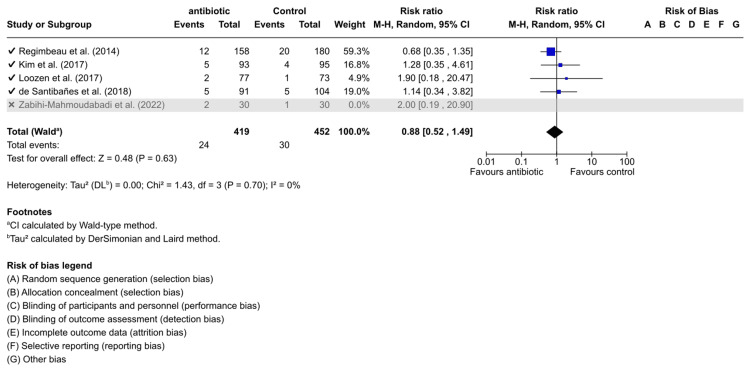
Sensitivity Analysis Results 1 (5). Regimbeau et al., (2014) [20], Kim et al., (2017) [18], Loozen et al., (2017) [19], de Santibañes et al., (2018) [13], Zabihi-Mahmoudabadi et al., (2022) [14].

**Figure 16:**
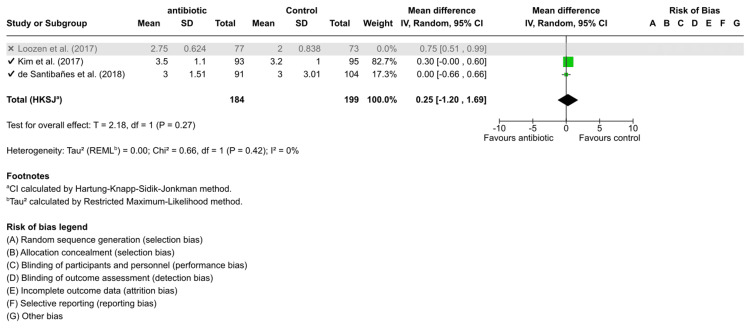
Sensitivity Analysis Results 2 (1). Kim et al., (2017) [18], Loozen et al., (2017) [19], de Santibañes et al., (2018) [13].

**Figure 17:**
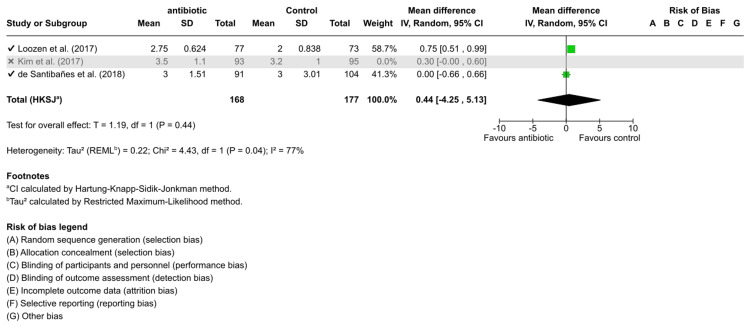
Sensitiity Analysis Resultsb2 (2). Kim et al., (2017) [18], Loozen et al., (2017) [19], de Santibañes et al., (2018) [13].

**Figure 18:**
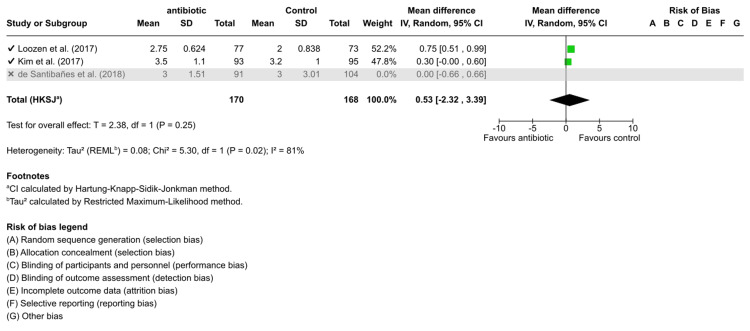
Sensitivity analysis 2 (3) Kim et al., (2017) [18], Loozen et al., (2017) [19], de Santibañes et al., (2018) [13]

## Appendices

Appendix

Sensitivity analysis (leave-one-out) for the surgical site infection forest plot (Figures 11-15).

Sensitivity analysis (leave-one-out), for the length of hospital stay, forest plot (Figures 16-18).
